# Potential value of preoperative fasting blood glucose levels in the identification of postoperative delirium in non-diabetic older patients undergoing total hip replacement: The perioperative neurocognitive disorder and biomarker lifestyle study

**DOI:** 10.3389/fpsyt.2022.941048

**Published:** 2022-10-13

**Authors:** Siyu Liu, Lizhu Xv, Xiaoyue Wu, Fei Wang, Jiahan Wang, Xinhui Tang, Rui Dong, Bin Wang, Xu Lin, Yanlin Bi

**Affiliations:** ^1^Department of Anesthesiology, Qingdao Municipal Hospital Affiliated to Qingdao University, Qingdao, China; ^2^Medical Department, Qingdao Municipal Hospital Affiliated to Qingdao University, Qingdao, China; ^3^Department of Anesthesiology, Dalian Medical University, Dalian, Liaoning, China; ^4^Department of Anesthesiology, Nanjing Medical University, Nanjing, China; ^5^Department of Anesthesiology, Gulou Hospital Affiliated to Medical College of Nanjing University, Nanjing, China

**Keywords:** preoperative fasting blood glucose, postoperative delirium, cerebrospinal fluid, biomarkers, cognitive

## Abstract

**Background:**

Postoperative delirium (POD) is a common complication after total hip replacement. This study aims to explore the relationship between preoperative fasting blood glucose (FBG) levels and POD in non-diabetic older patients undergoing total hip replacement.

**Materials and methods:**

This study included a total of 625 patients undergoing elective total hip replacement under combined spinal and epidural anesthesia from the PNDABLE study. The relationship between POD and preoperative FBG was analyzed by using the logistic regression model. The associations of FBG with individual cerebrospinal fluid (CSF) biomarkers were detected by using the multivariable linear regression model controlling for age, gender, and education level. The mediation effects were explored by mediation analyses with 5,000 bootstrap iterations, while sensitivity analysis was used to test the reliability and stability of the results. The receiver operating characteristic (ROC) curve and the nomogram model were applied to evaluate the efficacy of FBG and POD-related CSF biomarkers in predicting POD. POD assessment was performed two times daily by a trained anesthesiologist at 9:00–10:00 am and 2:00–3:00 pm on postoperative days 1–7 or before the patients were discharged from the hospital. POD was defined by the Confusion Assessment Method (CAM), and POD severity was measured using the Memorial Delirium Assessment Scale (MDAS). Enzyme-linked immunosorbent assay (ELISA) was used to measure CSF Aβ_40_, Aβ_42_, T-tau, and P-tau levels.

**Results:**

POD was detected in 10.2% (60/588) of the patients. Logistic regression analysis showed that after adjusting for age and education level, the increased levels of FBG (OR 1.427, 95% CI 1.117–1.824, *P* = 0.004), CSF P-tau (OR 1.039, 95% CI 1.019–1.060, *P* < 0.001), and CSF T-tau (OR 1.013, 95% CI 1.009–1.018, *P* < 0.001) were risk factors for POD, and the increased level of CSF Aβ_42_ (OR 0.996, 95% CI 0.994–0.998, *P* = 0.001) was a protective factor for POD. Multivariable linear regression models showed that when adjusting for age, gender, and education level, in the POD group, higher preoperative FBG levels were negatively correlated with the CSF Aβ42 level (β = −0.290, *P* = 0.028) and positively correlated with CSF P-tau (β = 0.384, *P* = 0.004) and T-tau (β = 0.447, *P* < 0.001). In the non-POD group, a higher preoperative FBG was not related to CSF biomarkers. Mediated effect analysis showed that CSF T-tau (proportion = 17.1%) had an apparent mediation effect on the relationship between FBG and POD. Sensitivity analysis revealed that the results from the logistic regression and multivariable linear regression models were consistent with previous results.

**Conclusion:**

Increased preoperative FBG was a risk factor for POD in older patients without T_2_DM, and T-tau might mediate the relationship between FBG and POD.

## Introduction

As defined in the Diagnostic and Statistical Manual of Mental Disorders fifth edition (DSM-5), postoperative delirium (POD) is a reversible neuropsychiatric disorder with disorganized thinking and altered consciousness, which possibly occurs on 1–7 days, particularly within 3 days postoperatively (1). POD, a frequent complication of elderly surgical patients, is related to significantly more extended hospital stays, cognitive impairment, functional decline, and increased mortality 6–12 months after surgery (2). With a rapidly aging population, the incidence of hip fracture continues to increase in China (3). One of the most common complications following hip fracture surgery is POD, and the incidence of POD after total hip replacement is approximately 20% (4–6).

More and more pieces of evidence support that type 2 diabetes mellitus (T_2_DM) is an independent risk factor for POD (7–9), and it increases the risk of POD in a variety of possible ways, including cerebrovascular diseases and neurodegenerative diseases (10). However, previous studies on the relationship between neuropathological outcomes and glucose metabolism mainly target the diabetic population (11). While studies investigating the effects of acute hyperglycemia in ICU patients found an association between hyperglycemia and delirium (12), the few available studies are primarily of a retrospective design and showed conflicting results (13, 14). The relationship between elevated preoperative FBG and POD is not clear.

Studies have shown that CSF biomarkers β-amyloid40 (Aβ_40_), β-amyloid42 (Aβ_42_), phosphorylated tau (P-tau), and total tau (T-tau) are associated with neurological abnormalities (15–17). These markers are usually associated with the pathogenesis of POD. Moreover, one study indicated that Aβ_42_ in CSF was positively associated with the T_2_DM status (18). So, we raise a question: In the preclinical stage of diabetes, is FBG as a risk factor for POD?

Therefore, the primary aim of this study is to explore the relationship between the occurrence of POD and preoperative FBG level in older patients without diabetes undergoing total hip replacement. The relationship between preoperative FBG and the POD CSF biomarkers (CSF Aβ_42_, Aβ_40_, T-tau, and P-tau) in older patients without diabetes undergoing total hip replacement was determined by our study to explore the possible underlying pathological mechanism of POD with the level of preoperative FBG using the mediated effect analysis. This study was the first to investigate the relationship between FBG and POD in non-diabetic patients undergoing total hip replacement. We hypothesized that patients with hyperglycemia are at higher risk for developing POD. In total, four analyses were performed. First, the relationship between POD and preoperative FBG was analyzed. Second, the relationship between FBG and POD CSF biomarkers was analyzed. Third, associations between FBG, POD CSF biomarkers, and POD were observed and analyzed. Finally, the stability and reliability of the results were tested.

## Materials and methods

### Ethical approval and study design

Non-diabetic cognitively normal northern Han Chinese participants were from the Perioperative Neurocognitive Disorder And Biomarker Lifestyle (PNDABLE) study. The PNDABLE is a large cohort study to analyze the risk factors and biomarkers of perioperative neurocognitive impairment in the Han population in northern China for the early diagnosis and prevention of the disease (19, 20). This study was approved by the Ethical Committee of Qingdao Municipal Hospital affiliated to Qingdao University, Qingdao, China (Ethical Committee N°2020 *PRO FORMA* Y number 005, date of bioethical committee approval: 2020. 05. 21), and written informed consent was obtained from all subjects participating in the trial. The trial was registered prior to patient enrollment at the China Clinical Trial Registry (clinical registration number: Chictr2000033439, Principal investigator: Yanlin Bi, date of registration: 2020. 06. 01). All patients were informed of the purpose and procedures (blood and CSF collection) of participation in the study, and informed consent was signed prior to enrollment.

### Study subjects

The study included a total of 625 patients undergoing elective total hip replacement under combined spinal and epidural anesthesia within the age-group of 40–90 years with class I–II American Society of Anesthesiologists (ASA) physical status classification, in the Qingdao Municipal Hospital affiliated to Qingdao University. The inclusion criteria of this study include patients (1) who were Han Chinese in north China; (2) with preoperative Mini-Mental State Examination (MMSE) scores of 23 or more; (3) with an educational level enough to the complete preoperative cognitive function test; and (4) with a good preoperative cognitive status with no language communication disorder. The exclusion criteria include (1) genetic family history; (2) central nervous system infection, head trauma, multiple sclerosis, epilepsy, and other major neurological diseases; (3) severe visual and hearing disorders; (4) major psychological dysfunction; (5) critical systemic diseases; (6) hypoglycemia; (7) diagnosed with diabetes; and (8) unwillingness to comply with the protocol or procedures.

**FIGURE 1:**
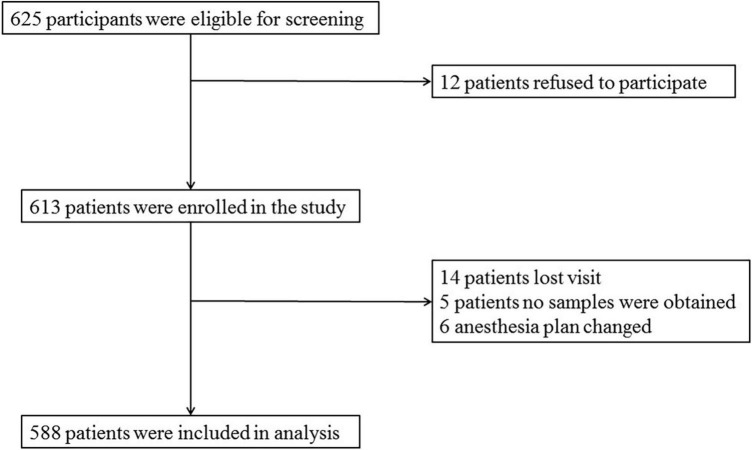
Diagram of study design.

Data of 588 patients were analyzed in this study, as shown in Figure 1.

### Neuropsychological testing

The neurologist used the MMSE on the day before the scheduled surgery. POD assessment was performed two times daily by a trained anesthesiologist at 9:00–10:00 am and 2:00–3:00 pm on postoperative days 1–7 or before patients were discharged from the hospital. POD was defined by using the Confusion Assessment Method (CAM), and POD severity was measured using the Memorial Delirium Assessment Scale (MDAS) (21, 22).

### Anesthesia and surgery

To avoid the influence of different surgical techniques, each patient undergoing elective total hip replacement were given combined spinal and epidural anesthesia by the same surgical team. The anesthesia position was lateral decubitus, with the space between the spinous processes of lumbar 3–4 (L3-L4) as the puncture site, and if the puncture fails, we will change the puncture site to be L2-3 or L4-5. After the successful puncture, 2 ml of CSF was extracted from the subarachnoid space, followed by an injection of 2–2.5 ml ropivacaine (0.66%) for about 30 s. After anesthesia, the sensory level was controlled below the thoracic 8 (T8) level. Electrocardiogram, pulse oxygen saturation, and non-invasive blood pressure were monitored continuously every 5 min during anesthesia. Midazolam was not allowed for intraoperative injection. During the surgery, oxygen was inhaled *via* a mask at 5 L/min to maintain blood pressure within ± 20% of the baseline value. If intraoperative NBP decreased below 90 mmHg (1 mmHg = 0.133kPa) or decreased by more than 20% of the baseline value, 5 mg ephedrine was injected intravenously. After the operation, the patient was sent to the post-anesthesia care unit (PACU), observed for 30 min, and sent back to the ward if there was no abnormality. Intravenous patient-controlled analgesia (butorphanol tartrate injection 10 mg + toranisetron hydrochloride injection 5 mg + 0.9% sodium chloride solution 89 ml maintained numeric rating scale < 3 points) was used in postoperative pain management. All clinical care details were recorded in the case report form.

### Fasting blood glucose and cerebrospinal fluid biomarker measurements

Blood samples were taken from patients after fasting for 8–10 h to test their FBG levels. FBG levels were measured by using the glucose hexokinase (HK) method.

Cerebrospinal fluid was collected according to the international consensus on standardization of CSF research (23) *via* a lumbar puncture in the L3/L4 or L4/L5 intervertebral space. The CSF sample was sent to the laboratory at room temperature within 2 h and centrifuged at room temperature for 10-min centrifugation (10 min, 1,800*g*, 4°C); CSF was stored in aliquots at −80°C until further analysis. CSF Aβ_42_, Aβ_40_, T-tau, and P-tau levels were measured by enzyme-linked immunosorbent assays (ELISAs) using INNOTEST (Fujirebio Europe N.V.). All CSF samples were distributed randomly across plates and measured in duplicate. All antibodies and plates were from the same batch to eliminate differences between batches. Intra-batch CV was <5%, and inter-batch CV was <15%. Experienced laboratory technicians blinded to clinical diagnosis and other clinical information conducted the experiments.

### Sample size estimation

The incidence of POD was 12%, the loss of follow-up was assumed to be 20%, and six covariates (age, education level, FBG, CSF Aβ42, T-tau, and P-tau) were expected to enter logistic regression, so the required sample size was calculated to be 625 cases (6 × 10÷0.12÷0.8 = 625).

### Data analysis

Continuous variables are presented as mean ± SD, and categorical variables as number (percentage). The chi-square test was used to compare the counting data. Two-independent sample *t*-test was used for the comparison of intra- and inter-group comparisons. Logistic regression was used to analyze the correlation between the FBG and POD after adjusting for age, education level, CSF Aβ42, T-tau, and P-tau levels. The odds ratio (OR) and 95% confidence interval (CI) of each factor were calculated. The associations of FBG with individual CSF measures were analyzed using multivariable linear regression models adjusting for age, gender, and educational level. All CSF variables in linear regression models were log-transformed to normalize the distributions and to facilitate comparisons between modalities. The mediated analysis was fitted according to the method proposed by Baron and Kenny to explore whether CSF biomarkers mediated the relationship between FBG and POD. The significance was determined by 5000 bootstrap iterations using the mediation effect. The indirect effect (IE) was *P* < 0.05, which was considered significant. Sensitivity analysis was used to test the stability and reliability of the results. All tests were two-sided. The statistical significance was set at *P* < 0.05.

StataCorp Stata MP 16.0 (Solvusoft Corporation, Inc, Chicago, IL, USA), R software version 4.1.1 (R Foundation for Statistical Computing, Vienna, Austria), and GraphPad Prism software, version 7.01 (GraphPad Software, Inc, LaJolla, CA, USA) were used for data analysis.

## Results

### Participant characteristics

A total of 625 patients were screened by inclusion and exclusion criteria, from which 37 were excluded (12 patients refused to participate, 14 patients were lost to follow-up, no samples were obtained from five patients, and six patients had anesthesia plans changed), and 588 non-diabetic cognitively normal patients remained for analysis. The demographic and clinical data of the participants are summarized in Table 1.

**TABLE 1:** Characteristics of participants.

	POD (*n* = 60)	Non-POD (*n* = 528)	*P*-value
Gender (male/female)	26/34	294/234	0.076
Age (year) (mean ± SD)	62.1 ± 9.54	60.3 ± 10.2	0.499
Body height (cm) (Median, IQR)	164.5 (160.0–171.5)	168 (160–173)	0.040
Body weight (kg) (Median, IQR)	69.5 (60.0–76.0)	70 (63–78)	0.335
BMI (mean ± SD)	25.5 ± 3.71	25.5 ± 3.67	0.931
Education year, n (%)			0.041
0	4 (6.7)	10 (1.9)	
1–9	33 (55)	272 (51.5)	
10–13	13 (20.5)	143 (27.1)	
14–17	10 (21.7)	103 (19.5)	
ASA grade, n (%)			0.663
I	9 (15.0)	91 (17.2)	
II	51 (85)	437 (82.8)	
Cigarette use, yes (%)	22 (36.7)	138 (26.1)	0.093
Alcohol intake, yes (%)	17 (28.3)	158 (29.9)	0.882
Hypertension, yes (%)	21 (35.0)	168 (31.0)	0.662
CHD, yes (%)	6 (10.0)	46 (8.7)	0.639
MMSE (Median, IQR)	28(27–29)	29(29–30)	0.374
Preoperative hemoglobin (Median, IQR)	129 (117.25–139)	133 (121–144)	0.160
Intraoperative blood loss (Median, IQR)	125 (110–135)	120 (110–130)	0.736
Intraoperative infusion volume (Median, IQR)	800 (800–900)	800 (800–900)	0.706
Duration of anesthesia (Median, IQR)	150 (130–160)	140 (130–160)	0.531
Duration of surgery (Median, IQR)	120 (110–130)	120 (110–130)	0.596
NRS scores (Median, IQR)	2 (1–2)	2 (2–2)	0.131
MDAS scores (Median, IQR)	11 (10–14)	3 (2–5)	< 0.001

Categorical variables are reported as numbers and percentages; continuous variables are reported as means ± SDs, whereas non-normal data are expressed as median (IQR). POD, postoperative delirium; BMI, body mass index; ASA, American Society of Anesthesiologists; min, minute; kg, kilogram; ml, milliliter; SD, standard deviation; IQR, interquartile range; CHD, coronary heart disease; MMSE, Mini-Mental State Examination; NRS, numeric rating scale; MDAS, Memorial Delirium Assessment Scale.

The incidence of POD we observed using the postoperative assessments was 10.2% (*n* = 60 of the 588 patients). In the POD group, participants were in their late adulthood (62.1 ± 9.54 years old) with a slight female predominance (66.7%). No significant differences were observed in age, sex, BMI, ASA grade, MMSE scores, preoperative hemoglobin, intraoperative blood loss, intraoperative infusion volume, anesthetic time, operative time, history of hypertension, and coronary heart disease between the POD group and the non-POD group.

### Comparison of fasting blood glucose and cerebrospinal fluid biomarker levels between two groups

**FIGURE 2:**
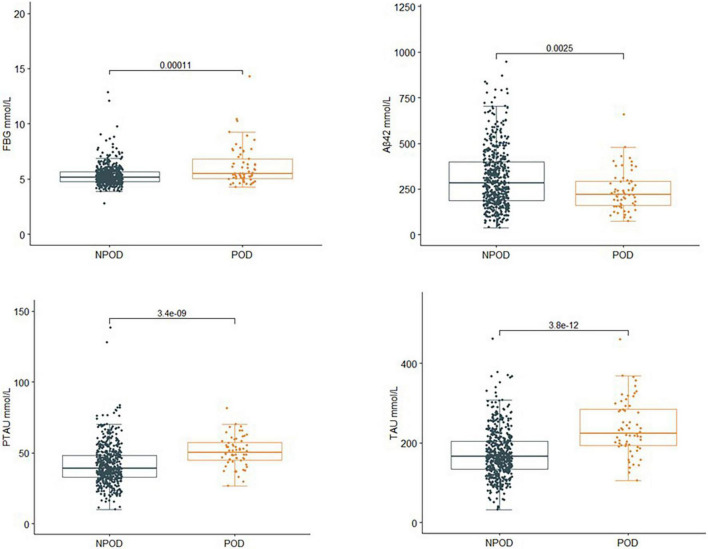
Comparison of FBG and CSF biomarker levels between two groups. Compared with the non-POD group, the differences in FBG (*P* < 0.001), CSF levels of P-tau (*P* < 0.001), and T-tau (*P* < 0.001) of the POD group were statistically significant (*P* < 0.05). POD, postoperative delirium; NPOD, no postoperative delirium; FBG, fasting blood glucose; Aβ42, β-amyloid42; T-tau, total tau; P-tau, phosphorylated tau.

The concentrations of FBG and CSF biomarkers (Aβ_42_, T-tau, and P-tau) were compared between POD patients and non-POD patients before the operation. Compared with the non-POD group, the differences in FBG (*P* < 0.001), CSF levels of P-tau (*P* < 0.001), and T-tau (*P* < 0.001) in the POD group were statistically significant (*P* < 0.05), as shown in Figure 2.

### Influencing factors of postoperative delirium by logistic regression

Univariate binary logistic regression analysis showed that increased concentrations of FBG (crude OR = 1.666, 95% CI 1.352–2.053, *P* < 0.001), CSF P-tau (crude OR = 1.040, 95% CI 1.022–1.059, *P* < 0.001), and T-tau (crude OR = 1.014, 95% CI 1.010–1.018, *P* < 0.001) were risk factors for POD. However, the increased concentration of Aβ_42_ (OR = 0.997, 95% CI 0.995–0.999, *P* = 0.002) was the protective factor of POD.

After adjusting for age and education level, the increased levels of FBG (OR 1.427, 95% CI 1.117–1.824, *P* = 0.004), CSF P-tau (OR 1.039, 95% CI 1.019–1.060, *P* < 0.001), and CSF T-tau (OR 1.013, 95% CI 1.009–1.018, *P* < 0.001) were risk factors for POD, and the increased level of CSF Aβ_42_ (OR 0.996, 95% CI 0.994–0.998, *P* = 0.001) was a protective factor for POD (Table 2).

**TABLE 2:** Logistic regression analysis.

Factors of interest	Unadjusted OR (95% CI)	*P*-value	Adjusted OR (95% CI)	*P*-value
Age (year)	1.008 (0.982–1.035)	0.541	1.017 (0.987–1.048)	0.258
FBG (mmol/ml)	1.666 (1.352–2.053)	< 0.001	1.427 (1.117–1.824)	0.004
Education level (year)	0.919 (0.857–0.985)	0.016	0.939 (0.870–1.014)	0.110
Aβ42 (pg/ml)	0.997 (0.995–0.999)	0.002	0.996 (0.994–0.998)	0.001
P-tau (pg/ml)	1.040 (1.022–1.059)	< 0.001	1.039 (1.019–1.060)	< 0.001
T-tau (pg/ml)	1.014 (1.010–1.018)	< 0.001	1.013 (1.009–1.018)	< 0.001

The factors of postoperative delirium (POD) were assessed using binary logistic regression analysis. OR, odds ratio; CI, confidence interval; FBG, fasting blood glucose; Aβ42, β-amyloid42; T-tau, total tau; P-tau, phosphorylated tau.

Sensitivity analyses were performed by (1) adding more covariates, including BMI, MMSE, cigarette use (yes or no), alcohol intake (yes or no), hypertension (yes or no), and coronary heart disease (yes or no). The results barely changed in this analysis, and (2) an MMSE ≥ 28 was used for screening patients.

### Relationship between postoperative delirium and biomarkers in cerebrospinal fluid by multivariable linear regression

**FIGURE 3:**
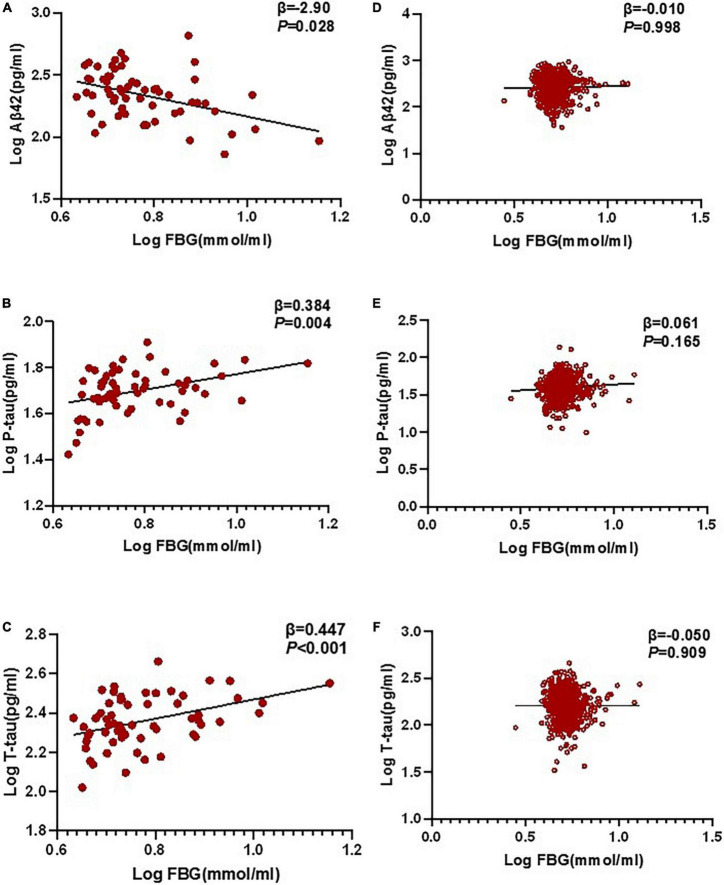
Relationship between POD and biomarkers in CSF by multivariable linear regression. In the POD group, higher preoperative FBG levels were negatively correlated with CSF Aβ42 level (β = –0.290, *P* = 0.028), and positively correlated with CSF P-tau (β = 0.384, *P* = 0.004) and T-tau (β = 0.447, *P* < 0.001) in the non-diabetic older patients **(A–C)**. In the N-POD group, higher preoperative FBG levels were not associated with CSF biomarkers **(D–F)**. FBG and all CSF variables in linear regression models were log-transformed to normalize the distributions. FBG, fasting blood glucose; Aβ42, β-amyloid42; T-tau, total tau; P-tau, phosphorylated tau.

In the multivariable linear regression model adjusted for age, gender, and education, the correlations between FBG and POD CSF biomarkers in CSF were tested. Multivariable linear regression models showed that when adjusting for age and gender, in the POD group, higher preoperative FBG levels were negatively correlated with the CSF Aβ42 level (β = −0.290, *P* = 0.028) and positively correlated with CSF P-tau (β = 0.384, *P* = 0.004) and T-tau (β = 0.447, *P* < 0.001) in the non-diabetic older patients (Figures 3A–C). In the non-POD group, higher preoperative FBG levels were not associated with CSF biomarkers (Figures 3D–F).

Sensitivity analysis outlying the mean ± 3 SD between CSF biomarkers was regarded as extremes and then excluded from the multivariable linear regression model; the results are consistent with those obtained previously.

### Associations between FBG, POD CSF biomarkers, and POD by mediated analysis

**FIGURE 4:**
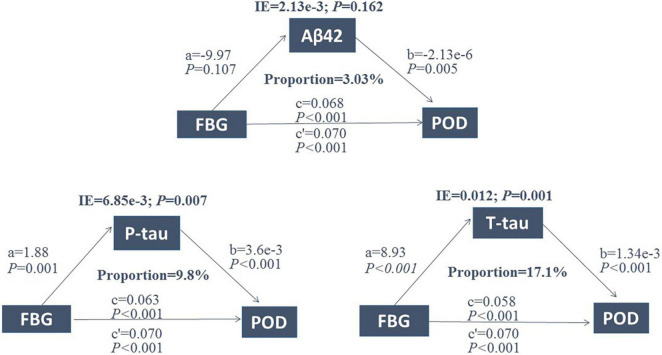
Mediation analysis. Relationship between FBG and POD was mediated by CSF T-tau (proportion = 17.1%). FBG, fasting blood glucose; POD, postoperative delirium; IE, indirect effect; Aβ42, β-amyloid42; T-tau, total tau; P-tau, phosphorylated tau.

Mediated effect analysis showed that CSF T-tau (proportion = 17.1%) significantly mediated the relationship between FBG and POD (Figure 4). These results suggested that FBG was not only a significant risk factor for POD but also a potential regulator of Tau pathology. Therefore, we further explored whether Tau pathology could mediate the effect of FBG on POD. The results showed that CSF T-tau might partly mediate the relationship between FBG and POD.

### Predictive model

**FIGURE 5:**
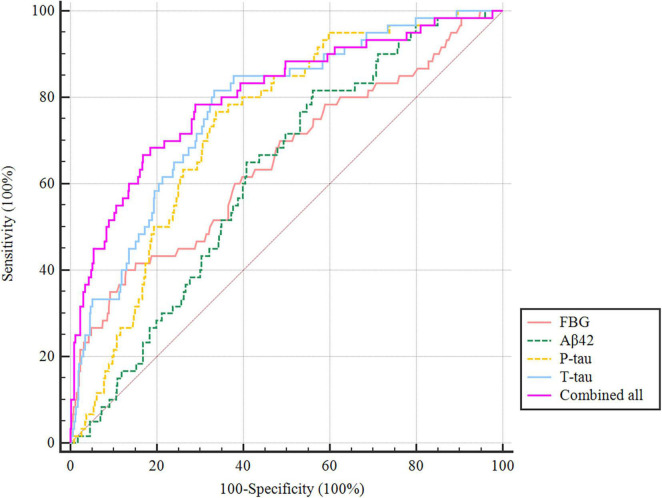
Predictive model. ROC curve showing the levels of FBG (AUC = 0.652; *P* < 0.001), CSF Aβ42 (AUC = 0.619; *P* < 0.001), P-tau (AUC = 0.733; *P* < 0.001), and T-tau (AUC = 0.773; *P* < 0.001) exhibited a discriminatory ability in POD prediction. POD, postoperative delirium.

**FIGURE 6:**
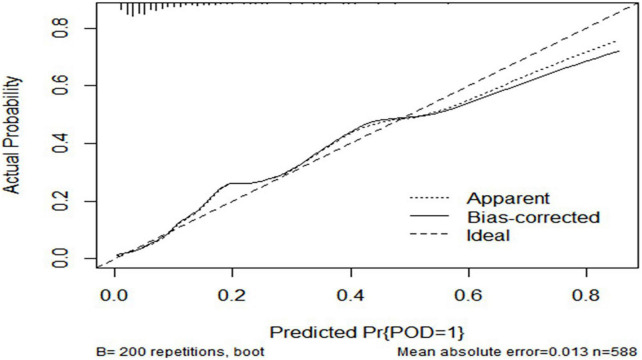
Predictive model. Calibration curve demonstrating the effectiveness of the predictive model. FBG, fasting blood glucose; Aβ42, β-amyloid42; T-tau, total tau; P-tau, phosphorylated tau.

**FIGURE 7:**
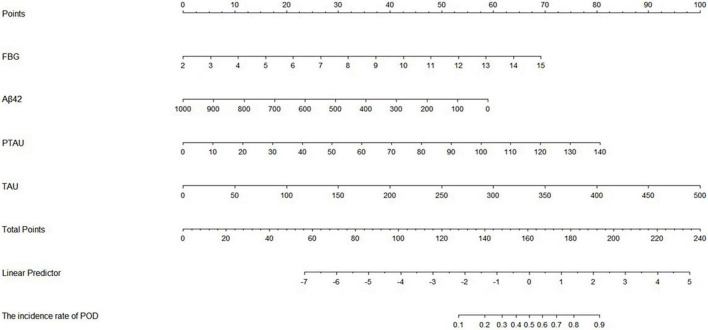
Visualization for predictive factors. Nomogram showing the predictive ability of FBG and the CSF biomarkers. AUC, area under the curve; FBG, fasting blood glucose; Aβ42, β-amyloid42; T-tau, total tau; P-tau, phosphorylated tau.

The ROC curve showed that the model combining FBG and CSF biomarkers (AUC = 0.803; *P* < 0.001) exhibited a relative better discriminatory ability in POD prediction compared with FBG only (AUC = 0.652; *P* < 0.001) (Figure 5), and the calibration indicated good prediction of it (Figure 6). The nomogram shows efficacy of each predictor (Figure 7).

## Discussion

In this prospective study, our study evaluated the association between preoperative FBG and POD in 588 patients who underwent total hip replacement under combined spinal-epidural anesthesia. Our study found that increased preoperative FBG is a risk factor for POD. CSF T-Tau mediated the relationship between FBG and POD.

The incidence of POD in this study was 10.2%, which was also consistent with the results of previous studies (24, 25). POD, an adverse postoperative complication, is the product of interacting basic disease, anesthetic factors, and surgical factors (26). Its pathogenesis is complex, including the theory of inflammatory response, the free radical, and theory of exogenous toxin damage, the theory of the cholinergic nerve, the theory of Tau protein hyperphosphorylation, and the theory of Aβ protein abnormal deposition.

In previous studies, biomarkers such as CSF Aβ_42_, T-tau, and P-tau can be vital for POD clinical research (27). T-tau has been considered a marker of gross neurodegeneration and axonal atrophy, and Tau protein abnormalities were found to be related to cognitive decline (28). Aberrant kinase–phosphatase coordination leads to an abundance of insoluble hyperphosphorylated tau aggregates. Hyperphosphorylated tau proteins disassociate from microtubules and interplay with other tau proteins to form tangles that disturb synaptic activity and neuronal signal (29, 30). The gradual accumulation of neurofibrillary tangles (NFTs) leads to neuronal degradation and inhibition of synaptic activity cascades, leading to sustained cognitive decline. In direct contact with the brain, CSF better reflects pathophysiological changes in the central nervous system (CNS); therefore, CSF is considered the best source of these biomarkers (31). This study included patients with selective spinal cord and epidural descending total hip replacement.

In non-diabetic patients, hyperglycemia has been found to lead to higher rates of adverse outcomes, including impairment of coagulation, cardiovascular events, and higher mortality rates (32–35). Furthermore, it is possible that hyperglycemia in non-diabetics is a marker for greater severity of illness or surgical stress than hyperglycemia in patients with diabetes. In fact, most previous studies found that T_2_DM poses a risk factor for POD. Both diseases share common pathological features: increased inflammation, oxidative stress, vascular damage, and glucose dysregulation (36, 37). Despite studies showing that T_2_DM may promote the early stage of neurodegenerative, the mechanisms remain clear. However, it is worth noting that the FBG levels were not high enough to develop pathological alterations in the non-diabetic population with the diabetes-like characteristics as described earlier.

This is the first population-based prospective study to assess the relationship between the incidence of POD and preoperative FBG levels and explore the relationships between FBG and CSF biomarkers in non-diabetic older patients. An increased FBG level, a risk factor for POD, was positively associated with P-tau and T-tau levels in cognitively normal patients without T_2_DM. In this study, univariate and multivariate logistic regression analyses were used to establish a POD clinical prediction model while drawing a nomogram in R language to visualize the clinical prediction model. In the present study, we discovered that CSF T-tau significantly mediated the relationship between FBG and POD. This result may give us a hint that FBG may mediate the occurrence of POD *via* CSF T-tau levels in an extent. Previous studies have shown that chronic hyperglycemia may lead to protein cross-linking and promote stabilization of the paired helical filament tau protein *via* increasing levels of advanced glycation end products (AGEs) (38). AGEs promote glycation of Aβ and Tau protein, respectively, leading to the formation of NFTs and aggregation of Aβ (39). In addition, increased glucose levels in brain tissue were associated with the deposition of Aβ and neurofibrillary pathological severity (40). The exact mechanism underlying how FBG can mediate POD remains unclear. This study proposes that elevated FBG in the preclinical stage of diabetes is a risk factor for POD. The result illustrates the effect of hyperglycemia on POD in non-T_2_DM patients and hints that elevated FBG in non-diabetic patients should be more valued in the perioperative period. In addition, we can guide the early screening of patients at a high risk of POD to provide a better reference for clinical work.

Some limitations should be acknowledged. First, this study was a single-center study, and we could carry out multi-center studies to further verify in future. Second, the number of patients was limited, and future studies will include more eligible patients.

## Conclusion

In conclusion, an elevated preoperative FBG level is one of the risk factors for developing POD in non-diabetic patients, and a predictive model based on the aforementioned indicators has good predictive efficacy. T-tau might mediate the relationship between FBG and POD. The study provided new perioperative management ideas for the prevention of POD. Early prevention of POD could improve patients’ quality of life, shorten hospital stays, and reduce family and social burdens.

## Data availability statement

The raw data supporting the conclusions of this article will be made available by the authors, without undue reservation.

## Ethics statement

The studies involving human participants were reviewed and approved by Ethical Committee Qingdao Municipal Hospital Affiliated to Qingdao University, Qingdao, China (Ethical Committee N°2020 *PRO FORMA* Y number 005). The patients/participants provided their written informed consent to participate in this study.

## Author contributions

SL was responsible for the design and implementation of this project, data collection, data statistics, and manuscript writing. BW conducted neuropsychological tests. RD was responsible for the patient data collection. XL and YB modified the manuscript. All authors have read and approved the final manuscript.
